# Response Properties of Electrorheological Composite Hydrophilic Elastomers Based on Different Morphologies of Magnesium-Doped Strontium Titanate

**DOI:** 10.3390/molecules29153462

**Published:** 2024-07-24

**Authors:** Shu-Juan Gao, Lin-Zhi Li, Peng-Fei Han, Ling Wang, Feng Li, Tan-Lai Yu, Yan-Fang Li

**Affiliations:** 1 Department of Chemical and Materials Engineering, Lyuliang University, Luliang 033001, China; 20121012@llu.edu.cn (L.-Z.L.); 20121013@llu.edu.cn (L.W.); 20081012@llu.edu.cn (F.L.); 20171017@llu.edu.cn (T.-L.Y.); 2 Institute of New Carbon-Based Materials and Zero-Carbon and Negative-Carbon Technology, Lyuliang University, Luliang 033001, China; 3 Institute of Teacher Education, Taiyuan Normal University, Taiyuan 030006, China; 201722801006@email.sxu.edu.cn

**Keywords:** Mg-doped strontium titanate, morphology, electrorheological composite hydrophilic elastomers

## Abstract

As smart materials, electrorheological elastomers (EREs) formed by pre-treating active electrorheological particles are attracting more and more attention. In this work, four Mg-doped strontium titanate (Mg-STO) particles with spherical, dendritic, flake-like, and pinecone-like morphologies were obtained via hydrothermal and low-temperature co-precipitation. XRD, SEM, Raman, and FT-IR were used to characterize these products. The results showed that Mg-STOs are about 1.5–2.0 μm in size, and their phase structures are dominated by cubic crystals. These Mg-STOs were dispersed in a hydrogel composite elastic medium. Then, Mg-STO/glycerol/gelatin electrorheological composite hydrophilic elastomers were obtained with or without an electric field. The electric field response properties of Mg-doped strontium titanate composite elastomers were investigated. We concluded that dendritic Mg-STO composite elastomers are high-performance EREs, and the maximum value of their energy storage was 8.70 MPa. The significant electrorheological performance of these products is helpful for their applications in vibration control, force transducers, smart structures, dampers, and other fields.

## 1. Introduction

Electrorheological (ER) materials have exhibited promise in the areas of new energy and new materials as actuators, valve devices, artificial muscles, flexible skins, etc. [1,2,3,4,5,6,7,8,9]. This is due to their controllability, reversibility, electric field responses (EFRs), and other electrorheological properties. Electrically and magnetically motivated smart composites include electrorheological fluids, magnetorheological fluids, electrorheological hydrogels, and magnetorheological elastomers. The rheology or animation viscoelasticity of such smart composites can be adjusted in real time and rapidly reversed by electric or magnetic fields. Thus, they are widely applied in vibration damping, variable rigidity control, and flexible robotics. The viscoelastic response properties of gels are sensitive to the internal structural changes in materials. Thus, their rheological behaviors are crucial for better understanding the flow properties and mechanical properties of ER materials.

Electrorheological elastomers are composite elastomers obtained by dispersing the electrolyte particles of the dispersed phase into an interpenetrating network (IPN) comprising a macromolecular compound [10,11,12,13,14,15,16]. These smart soft materials have stimulation responses to electric fields based on electrorheological fluids. EREs combine the advantages of electrorheological fluids and elastomers and can overcome the shortcomings of ordinary hydrogels, including facile precipitation and weak stability. The dispersed particles in EREs form specific anisotropic structures under electric fields, which significantly changes the mechanical modulus and shows potential for applying electric-field-modulated wave-absorbing materials in other areas [17,18,19,20]. However, high ERE rheological activity in similar electrorheological fluid levels has become a major challenge.

Recently, Shiga and coworkers prepared composite hydrogels by dispersing cobalt polyacrylate, a semiconducting polymer particle, in silica gel via cross-linking polydimethylsiloxane polymerization [21]. In their work, the energy storage shear modulus and loss modulus of the hydrogel increased, and the loss angular tangent changed with an applied DC electric field at room temperature [22]. Hanaoka and coworkers used hydrogenated methylsiloxane and ethylene methylsiloxane prepolymers containing unsaturated groups to obtain a continuous-phase polymer after a silica–hydrogenation polymerization reaction. To prepare a continuous phase with suitable elasticity and conductivity, the researchers chose prepolymers with particular physical parameters. The modulus magnitudes of the whole gel were modulated using the gelation temperature and time [23]. Shaw et al. obtained a series of silicone rubbers with different compositions by cross-linking two siloxanes in the presence of platinum catalysts [24]. They prepared speed-induced extensibility elastomers (SIEs) with good resilience and high toughness [25]. These thermoplastic elastomers had unique properties, including a positive correlation between the strain rate and the elongation of ruptures, moduli, and strength at room temperature. The elastomers exhibited excellent resilience, crack resistance, and self-healing properties. Joseph designed an electrorheological hydrogel actuator for a rehabilitation robot that could be applied to late-stage stroke treatment [26]. The actuator had a simple structure, a short response time, and low inertia, which are conducive to miniaturizing and domesticating rehabilitation robots. The results offered the possibility of applying electrorheological hydrogels in smart organs. All of the above studies showed that the composition and structure of the dispersed phase play an important role in influencing the electrorheological properties of hydrogels [27,28,29,30,31,32].

Given this research, the problems related to hydrophilic EREs have become a new hotspot in the electrorheological (ER) fluid material field. The electrical performance of ER particles and EFR particles depends on their polarization mode under an electric field [11,13,16]. ER properties are usually as good as EFR properties regarding easily polarizable particles. Particles with good EFR properties are a prerequisite for qualified ER particles. Thus, selecting a high dielectric constant dispersed phase is crucial for improving EFRs. Titanate doping with Mn, Fe, Co, etc., has been proven to exhibit excellent magnetodielectric and multiferroic response behavior. Mg acts as a chemical flavoring, which means that Mg-doped SrTiO_3_ has higher ER activity [33,34,35]. Cubic-magnesium-doped strontium titanate is the ideal dispersed phase material because of its high dielectric constant and strong ferroelectric response [36,37,38]. Magnesium-doped strontium titanate (Mg-STO) has the positive properties of magnesium and strontium titanate. Mg-doped strontium titanate particles may have a stronger EFR owing to the tendency of cubic strontium titanate to polarize spontaneously along the c-axis [39]. The polarity of SrTiO_3_ can be modified by doping it with different amounts of Mg, which, in turn, can improve its dielectric properties. Until now, the relationship between the morphology and EFR performance of Mg-doped SrTiO_3_ has rarely been investigated. Therefore, preparing Mg-doped SrTiO_3_ with different morphologies and studying its electric field response properties are important for theoretical and practical applications.

Thus, we prepared four different Mg-STO morphologies via the hydrothermal and low-temperature co-precipitation methods. The effect of morphology on the EFR performance of Mg-doped SrTiO_3_ is discussed to obtain high-performance dendritic Mg-doped SrTiO_3_ composite elastomers.

## 2. Results and Discussion

### 2.1. ICP Analyses of Mg-STO

**Figure 1:**
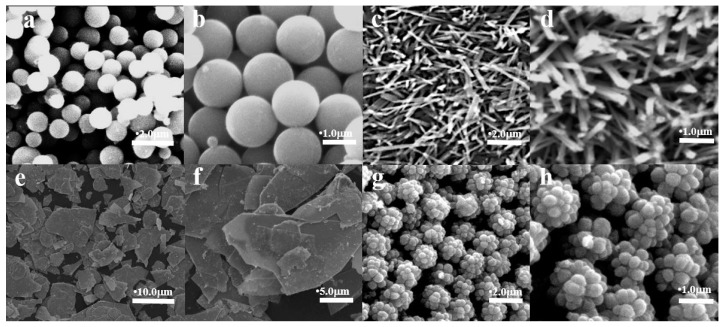
SEM patterns of Mg-STO: (**a**,**b**) spherical, (**c**,**d**) dendritic, (**e**,**f**) flake-like, and (**g**,**h**) pinecone-like.

Figure 1a,b shows SEM images of spherical samples. They are spherical with a regular shape and size of about 1.8 μm. A single dendritic Mg-STO comes in the form of an elongated long rod, and the length-to-diameter ratio of this “microrod” is large (Figure 1c,d). The diameter of dendritic SrMg*_x_*Ti_1−*x*_O_3_ is only a few nanometers, and the length is ~1 μm. Each branch consists of many small strips. Figure 1e,f shows the microstructure of flake Mg-STO, consisting of a group of sheet cells arranged haphazardly together. The particle size ranges from 5 μm to 20 μm. Figure 1g,h shows Mg-STO with a morphology similar to pinecones, with micrometer-sized particles formed by dozens of nanometer-sized balls [39]. That is, the micro–nanostructure is constructed by combining nano-sized matrix units. It is also the case that protruding spheres of about 300 nm in Figure 1h synergistically build micrometer-sized particles. The final micro-nanostructure reaches 2.0 μm.

Mg^2+^ (0.065 nm) has a similar radius to Ti^4+^ (0.068 nm), but it has a different radius from Sr^2+^ (0.135 nm). According to crystal chemistry theory, it can be inferred that Mg^2+^ can substitute for the position of Ti^4+^ in STO crystal lattices. The atomic radius of Mg is smaller than Sr, resulting in mostly smaller Mg-STO particle sizes [40]. The elemental compositions of samples M1–M4 were determined via ICP: Sr_1.10_Mg_0.03_Ti_1.00_O_3_, Sr_1.01_Mg_0.02_Ti_0.98_O_3_, Sr_1.02_Mg_0.02_Ti_0.98_O_3_, and Sr_1.07_Mg_0.02_Ti_0.99_O_3_, respectively (Table 1).

### 2.2. XRD and Raman Analyses of Mg-STO

**Figure 2:**
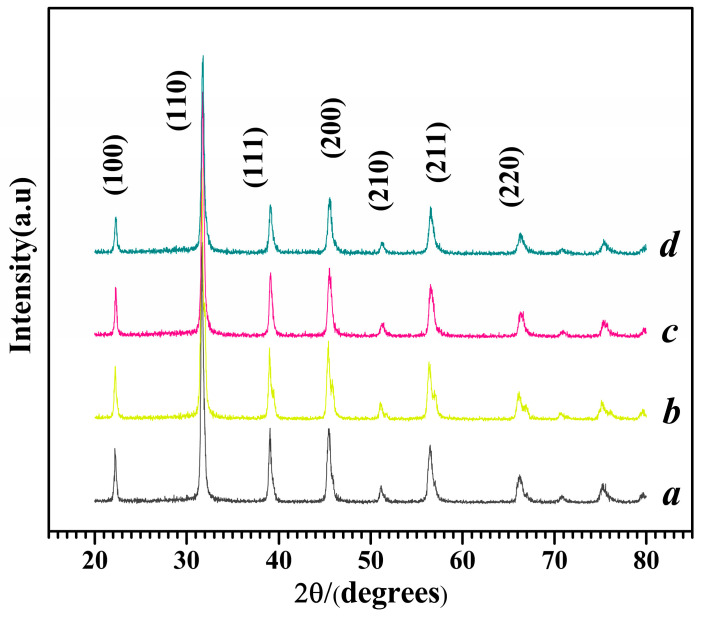
XRD patterns of different Mg-STO morphologies: (a) spherical, (b) dendritic, (c) flake-like, and (d) pinecone-like.

The X-ray diffraction (XRD) patterns of M1–M4 are shown in Figure 2, and the characteristic diffraction peaks of cubic Mg-STO have completely formed. The diffraction peaks of the four products are located at 31.35~32.42°, 22.1°, 31.5°, 38.7°, 45.1°, 50.7~56.0°, and 66°, corresponding to the seven characteristic peaks in the Mg-doped SrTiO_3_ standard card (JCPDS No. 073-0661; the error in the XRD angle was ~0.02°). The diffraction peaks of the four samples were almost identical to the standard peaks, except for weak shifts at [110] and [211]. This suggests the partial substitution of Ti^4+^ by Mg^2+^ in SrTiO_3_. The survey XPS spectra further verified the successful Mg to Ti species ion exchange (Figure S2, Supporting Information (SI)). This agrees with the above XRD results. Moreover, small shifts toward lower binding energy in the Mg 1s and O 1s peaks and a pronounced shift toward higher binding energy in the Ti 2p peaks can be identified, and these can be ascribed to the electronic interaction between the incorporated Mg^2+^ and TiO_6_ layers (Figure S3, SI).

**Figure 3:**
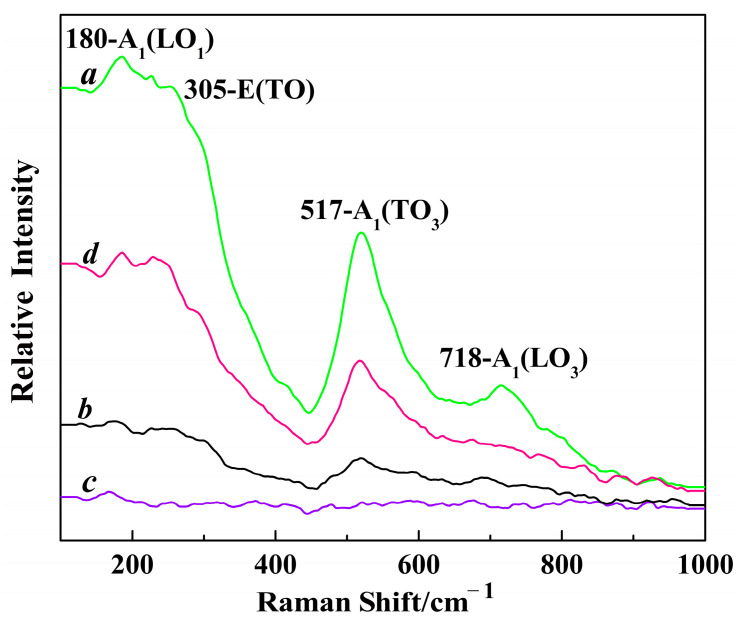
Raman patterns of different Mg-STO morphologies: (a) spherical, (b) dendritic, (c) flake-like, and (d) pinecone-like.

Raman techniques were employed to further accurately characterize the crystalline phase of the Mg-STO. The 305 cm^–1^ neighborhood band corresponds to the Bl vibrational mode in Figure 3 [41]. This is the characteristic band of tetragonal Mg-STO. The 305 cm^–1^ band begins to appear at step one in the dendritic and spherical patterns, which indicates a tetragonal structure. The transverse optical phonon modes of A_1_(TO_1_), A_1_(TO_2_), and A_1_(TO_3_) correspond to 155, 260, and 518 cm^–1^, respectively. The Raman activity peaks appear near 718 cm^–1^ for A_1_(LO_3_). E(TO_2_) corresponds to almost 305 cm^–1^ and is the characteristic peak of the tetragonal phase. By contrast, the tetragonal characteristic peaks are weak or disappear in the flake-like and pinecone-like patterns. This is because the number of tetragonal structures is tiny and almost undetectable. This also means that the products have no Raman activity; i.e., no diffraction peaks corresponding to the products of the tetragonal phases appeared in the Raman patterns. Hence, they belong to the cubic phase.

The four Mg-STOs were dominated by a cubic–perovskite phase structure, consistent with our XRD observations. The Raman activity intensities of the four samples were in the following order: spherical > pinecone-like > dendritic > flake-like. Polarization occurs in the interior of the crystal where Mg^2+^ and Sr^2+^ ionic layers alternate along the [001] direction, creating microelectric domains. Subsequently, a dipole moment is generated, which shifts the polarity of the particles. However, these polarity shifts only occur in local regions; these phenomena are known as lattice structure micro-disturbances. They hardly affect the basic structure of the crystals, and this is why Mg-doped STOs differ in microstructure from STOs with classical perovskite structures.

### 2.3. FI-IR Analyses of Mg-STO

**Figure 4:**
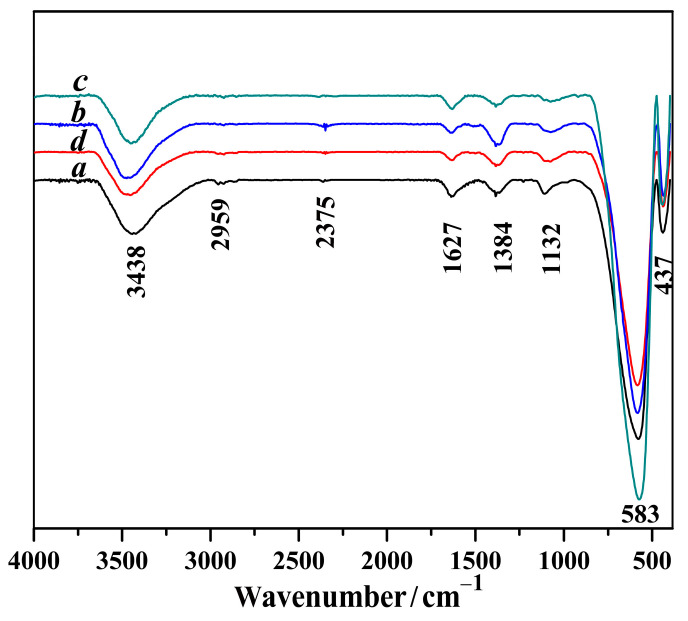
FT-IR diagrams of four kinds of Mg-STO morphologies: (a) spherical, (b) dendritic, (c) flake-like, and (d) pinecone-like.

Figure 4 illustrates the stretching vibrations, *v*(O–H), of the O–H broadband at 3438 cm^−1^ for Mg-STO [42]. The two weak absorption peaks that appear near 2900 cm^−1^ are C–H stretching vibrations. The weak vibrational absorption peak at 2400 cm^−1^ can be attributed to the characteristic peak of uncleaned SrCO_3_. A spectral band near 1627 cm^−1^ can be assigned to the coordinated water of H–O bending vibrations [43]. Two sharp peaks at 1300~1100 cm^−1^ are evidence of C–H bending vibrations in the alkyl chain, suggesting the presence of minor residual ethanol on the surface of the Mg-STO. The FTIR results show that the Ti–O stretching vibration bands are around 400~1000 cm^−1^ in the products. In contrast to the normal 612 cm^−1^ band, this broadband is a stretching mode of a Ti–O bond in TiO_2_. The bands at 583 cm^−1^ and 437 cm^−1^ are generated by the Ti–O stretching vibrations in the four Mg-STOs. The intensity and sharpness of these absorption bands increase dramatically, indicating that all four products are pure Mg-STO crystals.

### 2.4. Hydrophilicity Measurement and BET Analyses of Mg-STO

**Figure 5:**
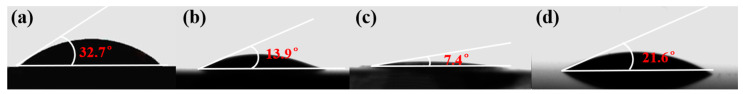
Contact angles of water drops (3 μL) on Mg-STOs with different shapes: (**a**) spherical, (**b**) dendritic, (**c**) flake-like, and (**d**) pinecone-like.

The infiltration on the surfaces of the M1–M4 particles was measured using the sessile drop method under a video-based optical contact angle meter. The current test was performed on Mg-doped STO thin film samples. It was prepared as follows: a suspension of dried Mg-STO in ethanol was prepared via grinding and ultrasonic dispersion. A clean glass slide was then inserted vertically and slowly pulled out at a set speed (5 cm/s). Finally, it was dried at room temperature, and the process was repeated three times. Then, Mg-STO films formed on the surface of the slide, and 3 μL of water was dropped into the film using a fine-tuned syringe. The contact angle between the waterdrop and the film was measured. The contact angles between the different Mg-doped STO morphologies and the water are shown in Figure 5. Unsurprisingly, the contact angles of M1–M4 with water were 32.7°, 13.9°, 7.4°, and 21.6°, respectively. They were all less than 35.0°, indicating that they are superhydrophilic materials with excellent dispersion and good compatibility in water [44,45,46].

All four products are superhydrophilic, given the small wetting angle observed (especially for flake-like particles), and the flake-like product has the highest hydrophilicity because the water penetration within the interparticle spaces is greater. As a supplementary verification, we determined the specific surface areas of the four products using the multiple-point Brunauer−Emmett−Teller (BET) method. Notably, the surface energies of the spherical, pinecone-like, dendritic, and flake-like products were 17.42, 18.53, 19.65, and 18.27 m^2^ g^−1^, respectively. Of these, dendritic structure had the largest surface energy, implying that the more reactive its surface is, the more easily it can be wetted and show better hydrophilicity. The large surface energy also means that the dendritic Mg-doped STO surface is prone to chemical reactions.

The hydrophilicity of the four Mg-STOs is in the following order: flake-like > dendritic > pinecone-like > spherical. According to the rule of surface roughness on the contact angle of microstructures, the larger the surface roughness, the smaller the contact angle. The order of roughness for the four products is flake-like > dendritic > pinecone-like > spherical. Therefore, the contact angle of flake-like Mg-STO is the smallest, and it thus has maximum hydrophilicity. We also calculated the surface energies of the four Mg-STOs. The surface energy test was performed using water and diiodomethane as a titrant. The dispersive and polar components of the surface energy of water–diiodomethane were 22.10/48.50 and 50.65/2.30 mN/m, respectively. The surface energies of the flake-like, dendritic, pinecone-like, and spherical Mg-STOs were calculated to be 72.41, 71.24, 68.81, and 64.07 mN/m using the T-Yong equation [47]. The flake-like Mg-STO had the largest surface energy, in line with its maximum hydrophilicity. The larger the surface energy, the stronger the wettability of the liquid owing to intermolecular van der Waals and Coulomb forces. Furthermore, the larger the proportion of polar parts in the dispersive and polar energies, the stronger their adsorption capacity.

### 2.5. Dielectric Measurement of Mg-STO

**Figure 6:**
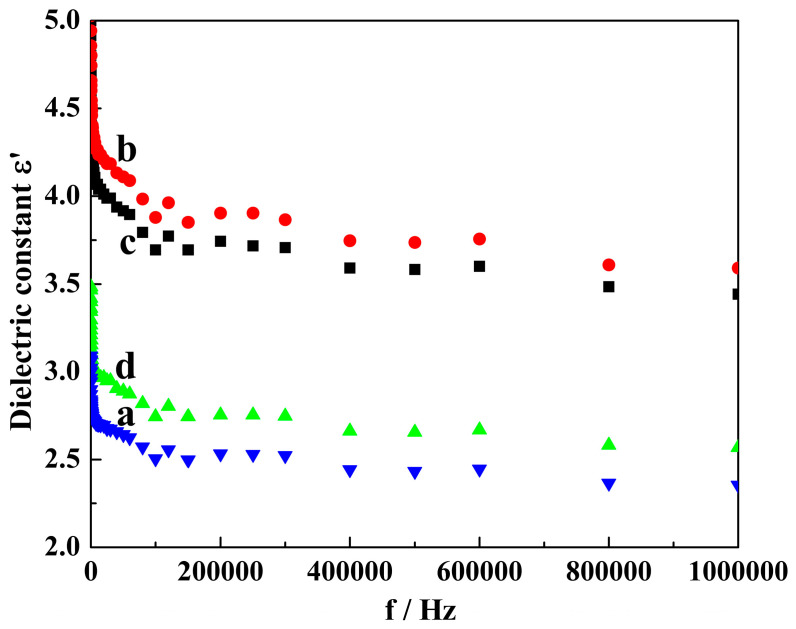
Dielectric spectra of the as-made samples: (a) spherical, (b) dendritic, (c) flake-like, and (d) pinecone-like.

The dielectric spectra of the Mg-STO ER fluids from 20 Hz to 1 MHz in frequency were experimentally investigated to determine the relationship between their structures and dielectric properties (Figure 6). Dried Mg-STO was dispersed into silicone oil (η = 50 mPa·s at 25 °C) through grinding and ultrasonic dispersion. The dielectric mass spectra of the samples were then measured by a current analyzer with a 1 V bias potential. Figure 6 shows the frequency dependence of the real part (ε′) of the complex dielectric constant. The room-temperature dielectric constants of all products were not significantly influenced by the frequency fluctuation (they *only slightly decreased*) [48]. The order of dielectric constant magnitudes for the four products is dendritic > flake-like > pinecone-like > spherical. Each has a maximum dielectric constant (6.15, 5.78, 3.49, and 3.09) at a particular frequency (20, 20, 25, and 20 Hz). We speculated that crystal dipoles move relatively easily under low DC voltage electric fields. When the particle size is determined, the wider the interplanar crystal spacing, the greater the tetragonal distortion of unit cells. The interplanar crystal spacing of the dendritic Mg-STO is larger than the other three morphologies; therefore, the dielectric constant of dendritic Mg-STO reaches the maximum value [49].

### 2.6. Analyses of EFR Performance of Mg-STO

The energy storage modulus is the amount of elastic energy stored per unit volume within the elastic deformation ranges of a material. It is one of the most important metrics for determining the elastic properties of elastomers. The energy storage modulus reflects the degree of elastic deformation of a material under stress, which can also be understood as the degree of softness or rigidity of the elastomer. The energy storage modulus is closely related to the elastic modulus of an elastomer. The elastic modulus is the ability to restore a material to its original state with elastic deformation, and it is a physical quantity that describes the stiffness of the elastomer. Typically, the energy storage modulus is half the elasticity modulus, which is equal to the elasticity modulus multiplied by half the coefficient of the elastomer’s volumetric elasticity.

**Figure 7:**
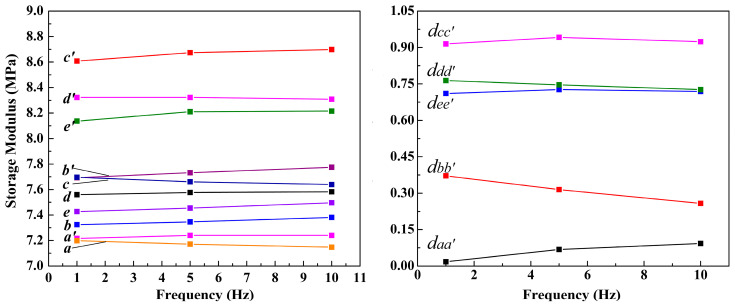
The energy storage modulus/frequency graphs of composite elastomers with different Mg-STOs under an electric field: (a~e) 0.0 kV/mm and (a’~e’) 1.2 kV/mm. a and a’: without Mg-STO; b and b’: with spherical Mg-STO; c and c’: with dendritic Mg-STO; d and d’: with flake-like Mg-STO; e and e’: with pinecone-like Mg-STO. The gap between curves a and a’ is labeled *d_aa’_*, and others are similar.

The energy storage moduli of the A- and B-elastomers (Mg-STO/glycerol/gelatin hydrogel elastomers) versus the frequency at a fixed strain of 0.01% are shown in Figure 7. The storage modulus values for each elastomer exhibit insignificant variations with increasing frequency. Figure 7 a and a’, respectively, represent elastomers without and with an applied electric field that does not contain dispersed particles. Figure 7 b(b’)~e(e’) represent, respectively, composite elastomers with and without an electric field, where 1.0 wt% of M1–M4 was added. Figure 7 a and a’ almost overlap, indicating that the pure gelatin–glycerol hydrogel elastomer had no obvious response performance without an electric field. The curves of b(b’)~e(e’) are located above a and a’; i.e., the energy storage modulus of the elastomer containing Mg-STO particles is larger than that of the pure elastomer at the same frequency. Remarkably, but not surprisingly, the hardness of the composite elastomer becomes larger owing to being filled with Mg-STO. In addition, modulus/frequency curves of b’, c’, d’, and e’ of the B-elastomers are above curves b, c, d, and e of the A-elastomers. This means that the resilience of the B-elastomers is greater than the A-elastomers, demonstrating the significant response performance of M1–M4.

We assumed that the dispersion particles are randomly distributed in the elastomer when there is no electric field present during the gelation process. Here, the Mg-STO only has a filling effect. Following this, the elastomer structure obtained is isotropic. However, the polarized Mg-STO can form a chain or columnar microstructure in the elastomer under an applied electric field. This enhances the elastomer’s compressive properties and increases its hardness [20,24,40,50]. Furthermore, we found that the spacing, *d_cc’_*, of curves c and c’ was larger than that of dd’, ee’, and bb’ (*d_cc’_* > *d_dd’_* > *d_ee’_* > *d_bb’_* > *d_aa’_*; Figure 7, right panel). The larger gap in the energy storage modulus/frequency curves implies better response behavior in the dispersed particles under an electric field. Consequently, a line of variation exists in the “high degree of polarization of the dispersed particles―regular and ordered aggregation in the electric field―increasing hardness of electrorheological composite elastomers―increasing difference of hardness between A- and B-elastomers” [40,41,51,52].

In conclusion, the ΔG values of the elastomers dispersed with dendritic and flake-like particles are much higher than others. This is because the dendritic Mg-STO particle has a high dielectric constant, and the flake-like particle has the best interface consistency owing to its significant hydrophilicity, so they are easily polarized under an electric field. As a result, particles with high dielectric constants and strong hydrophilicity are polarized under an electric field, enhancing the energy storage modulus of the elastomer and providing it with a strong electrical response.

## 3. Materials and Methods

### 3.1. Preparation of Mg-STO

#### 3.1.1. Preparation of Spherical and Pinecone-Shaped Mg-STO

Spherical and pinecone-shaped Mg-doped SrTiO_3_ samples were labeled M1 and M4, respectively. They were prepared with a one-step hydrothermal method using titanium tetrachloride as the titanium source, sodium hydroxide as the mineralizing agent, and dilute hydrochloric acid as the hydrolysis inhibitor [40]. The morphology of M1 was spherical; it was monodispersed and regular, with a smooth surface and a size of about 1.8 μm (*vide infra*). M4 had a pinecone-shaped micro-nanostructure, composed of dozens of nano-sized spheres with a size distribution of 2.0 μm (vide infra).

#### 3.1.2. Preparation of Dendritic and Flake-like Mg-STO

Based on methods in the literature [53], Mg-doped SrTiO_3_ samples with dendritic and flake-like morphologies were prepared via a low-temperature co-precipitation method by regulating the pH of a SrMg*_x_*Ti_1−*x*_O(C_2_O_4_)_2_ precursor solution. In contrast to the literature, Sr(OH)_2_ and Mg(NO_3_)_2_ were added to a substituted B-site based on a solid solubility limit of about 2.00 mol%, i.e., *n*[Sr^2+^]:*n*([Mg^2+^]+[Ti^4+^]) = 1.00, *n*[Mg^2+^]:*n*([Mg^2+^]+[Ti^4+^]) = 0.02. Remarkably, dendritic Mg-doped SrTiO_3_ M2 was obtained with a pH = 4.97 SrMg*_x_*Ti_1−*x*_O(C_2_O_4_)_2_ precursor solution. Flake-like Mg-doped SrTiO_3_ M3 was followed by a SrMg*_x_*Ti_1−*x*_O(C_2_O_4_)_2_ precursor solution with pH = 6.87. The final products were then calcined in a muffle furnace (vacuum) at 700 °C for 4 h.

### 3.2. Preparation of Electrorheological Composite Hydrophilic Elastomers

A specific amount of Mg-doped SrTiO_3_ was ground, homogenized, and dispersed in gelatin–glycerol–water at 65 °C in a water bath. The cross-linking agent glutaraldehyde was quickly added and placed in two Plexiglas boxes (40 × 20 × 3 mm^3^). The gel was applied in the presence or absence of an external DC electric field (1.2 kV/mm) for 30 min at 65 °C and moved to room temperature for 20 min. Finally, the gelation was maintained for 7 h to obtain an elastomer without an external electric field. Mg-doped SrTiO_3_ gelatin and glycerol hydrophilic elastomers were prepared, herein referred to as the A-elastomers (0.00 kV/mm) and B-elastomers (1.20 kV/mm), respectively [53,54,55]. The energy storage moduli of the composite elastomers were tested using dynamic viscoelastic spectroscopy, and the energy storage modulus/frequency curves were obtained in multi-frequency modes. The relationship between the hardness variation in the composite elastomer and the morphology of the Mg-STO was explored based on its curves [53,56]. Thus, we were able to further investigate the exotic response properties of Mg-STO samples with different morphologies when applying electric fields [40].

### 3.3. Characterizations

The morphologies and structures of the products were studied using a scanning electron microscope (SEM, (Questar, New York, NY, USA), 450 Quanta™(Thermo Fisher Scientific Inc., New York, NY, USA)) and a Fourier transform infrared spectrometer (FTIR, EQUIOX55 (Bruker, Billerica, MA, USA), in a range of 4000–500 cm^−1^). X-ray diffraction spectra were recorded using a diffractometer (XRD, Rigaku D/MAX-2550 (Rigaku, Tokyo, Japan), λ = 1.5512 Å) for Cu/Kα radiation. The elemental composition of the product was detected by an ICP-AES tester. The contact angle of the Mg-STO was measured by an OCA20 video optical contact angle tester (Dataphysics, Charlotte, NC, USA). The pH of the solution was checked with a German Sedovis PB-10 acidity meter (Sedovis, Gottingen, Germany). Finally, the dielectric properties were obtained with an HP4284A dielectric constant tester (Agilent, Santa Clara, CA, USA). The energy storage modulus of the composite elastomer was eventually measured by a Q800DMA dynamic viscoelastic spectrometer(TA, New Castle, DE, USA).

## 4. Conclusions

In this study, four SrMg*_x_*Ti_1−*x*_O_3_ samples with different morphologies (spherical, dendritic, flake-like, and pinecone-like) were successfully prepared via two methods: the one-step hydrothermal method and the low-temperature co-precipitation method. These samples had different compositions, surface morphologies, surface hydrophilicity, and dielectric properties. The rough surface morphology of cubic Mg-doped SrTiO_3_ affects its surface hydrophilicity, and particles with higher length–diameter ratios exhibit better dielectric properties. The four products were dispersed in a hydrogel composite elastomer medium. Then, the EFR properties of these Mg-STO/glycerol/gelatin composite elastomers were investigated. In conclusion, the high cubic structure, excellent dielectric properties, and super hydrophilicity of dendritic Mg-doped SrTiO_3_ particles have a synergistic effect on the electric field response performance of electrorheological composite hydrophilic elastomers. We confirmed that the molecular formulae of the M1–M4 samples were Sr_1.10_Mg_0.03_Ti_1.00_O_3_, Sr_1.01_Mg_0.02_Ti_0.98_O_3_, Sr_1.02_Mg_0.02_Ti_0.98_O_3_, and Sr_1.07_Mg_0.02_Ti_0.99_O_3_, respectively. By measuring contact angles, we found that these four kinds of magnesium-doped strontium titanate were superhydrophilic materials, and all contact angles were less than 33.0°. The EFR properties of the samples were in the order dendritic > flake-like > pinecone-like > spherical, which is consistent with the arrangement of dielectric constants. Eventually, we determined that the maximum energy storage modulus and dielectric constant values were 8.70 MPa and 6.15, respectively.

We established and developed a simple, efficient, low-cost pathway for producing electrohydrophilic hydrogels based on designing and preparing the functional materials of Mg-doped strontium titanate/gelatin/glycerol hydrophilic elastomers. This promotes the efficient use of smart and medical biomimetic materials in the field.

## Figures and Tables

**Table 1:** ICP analysis data for Mg-STO.

Sample	Sr/mol	Mg/mol
M1	1.095 ± 0.002	0.030 ± 0.002
M2	1.013 ± 0.001	0.017 ± 0.001
M3	1.021 ± 0.001	0.023 ± 0.001
M4	1.071 ± 0.001	0.022 ± 0.001

## Data Availability

Data are contained within the article and supplementary materials.

## Supplementary Materials

The following supporting information can be downloaded at https://www.mdpi.com/article/10.3390/molecules29153462/s1: Figure S1: Combined graph showing maximum-resolution SEM patterns and energy storage modulus–frequency patterns for different Mg-doped STO/glycerol/gelatin electrorheological composite hydrophilic elastomer morphologies; Figure S2: XPS survey spectra of different morphologies of Mg-STO (a) spherical, (b) dendritic, (c) flake-like, and (d) pinecone-like; Figure S3: Ti 2p XPS spectra of different morphologies of Mg-STO (a) spherical, (b) dendritic, (c) flake-like, and (d) pinecone-like.
