# Supplementary material for: Response Properties of Electrorheological Composite Hydrophilic Elastomers Based on Different Morphologies of Magnesium-Doped Strontium Titanate
Source: Molecules. 2024 Jul 24;29(15):3462. doi: 10.3390/molecules29153462 (PMC11313832; doi:10.3390/molecules29153462)

SUPPLEMENTARY INFORMATION

Response Properties of Electrorheological Composite Hydrophilic Elastomers Based on Different Morphologies of Magnesium-Doped Strontium Titanate

Shu-Juan Gao [1,2,*], Lin-Zhi Li [1,2], Peng-Fei Han [3], Ling Wang [1], Feng Li [1], Tan-Lai Yu [1,2] and Yan-Fang Li [1,*]

[1]*Department of Chemical and Materials Engineering, Lyuliang University, Lishi Shanxi 033001, China; shujuangao@llu.edu.cn (S.-J.G.)*

[2]*Institute of New Carbon-based Materials and Zero-carbon and Negative-carbon Technology, Lyuliang University, Lishi Shanxi 033001, China; shujuangao@llu.edu.cn (S.-J.G.)*

[3]*Institute of Teacher Education, Taiyuan Normal University, Taiyuan 030006, China; 201722801006@email.sxu.edu.cn (P.-F.H.)*

* Correspondence: shujuangao@llu.edu.cn; 20131017@llu.edu.cn

Supplementary Information

Figure S1. The storage modulus-frequency of the composite elastomers with different Mg-STO (a)~(e) 0 kV/mm, (a')~(e') 1.2 kV/mm, a a': without Mg-STO, b b': with spherical, c c': with dendritic, d d': with flake-like, and e e': with pinecone-like. The small panel at the upper left part show the maximum scanning resolution graphs corresponding to the four morphologies. The small panel at the upper right part shows the relative values of differences between the charged and uncharged modulus of energy storage for four shape products.

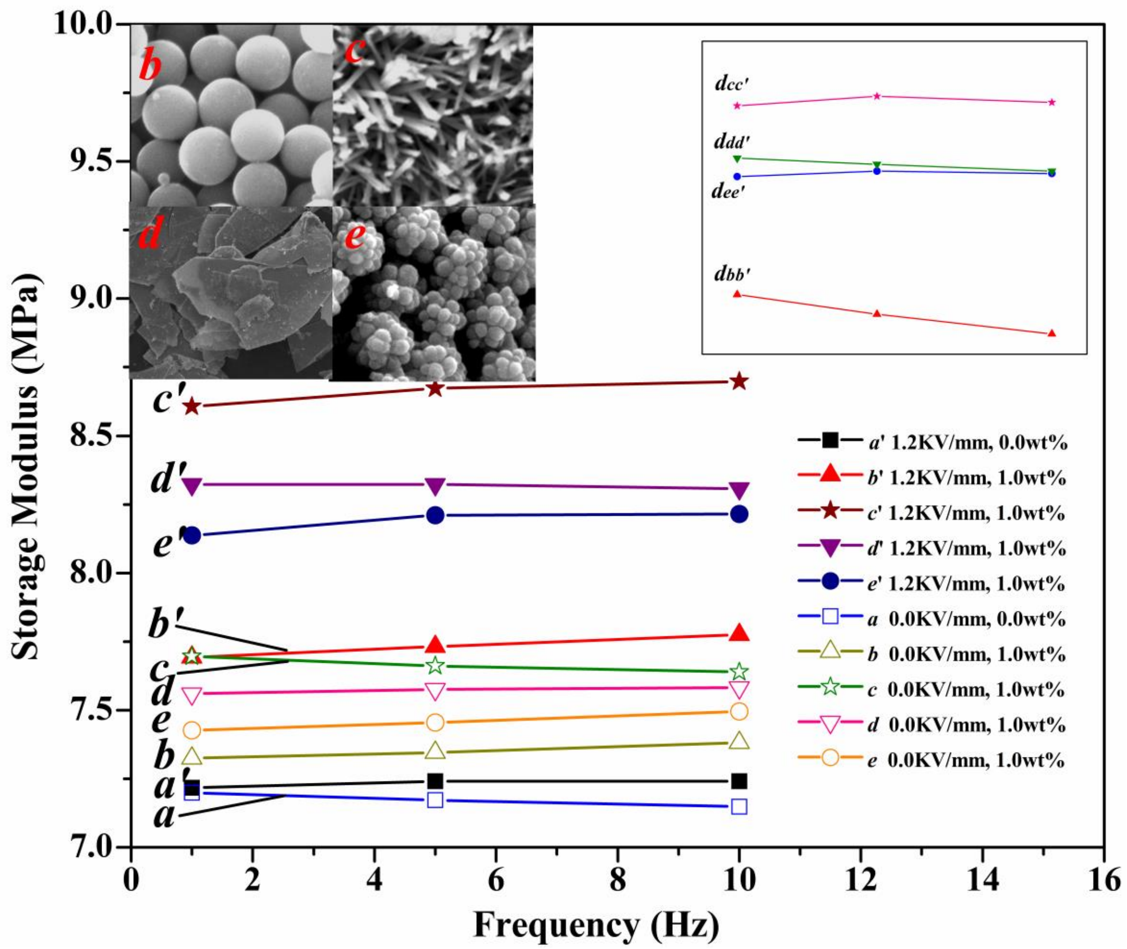

Figure S2. XPS survey spectra of different morphologies of Mg-STO (a) spherical, (b) dendritic, (c) flake-like, and (d) pinecone-like.

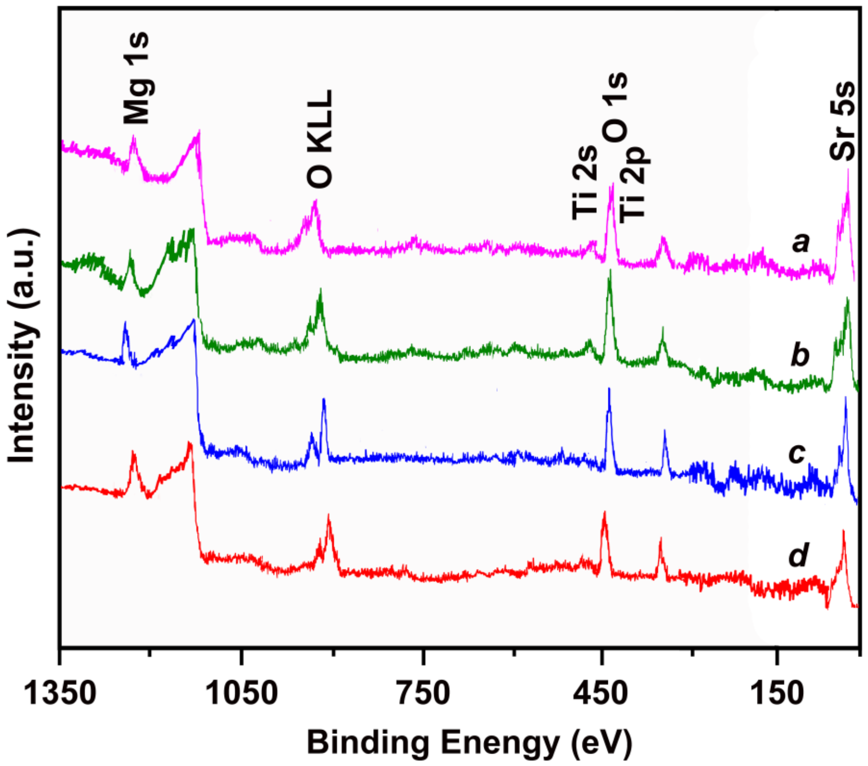

Figure S3. Ti 2p XPS spectra of different morphologies of Mg-STO (a) spherical, (b) dendritic, (c) flake-like, and (d) pinecone-like.

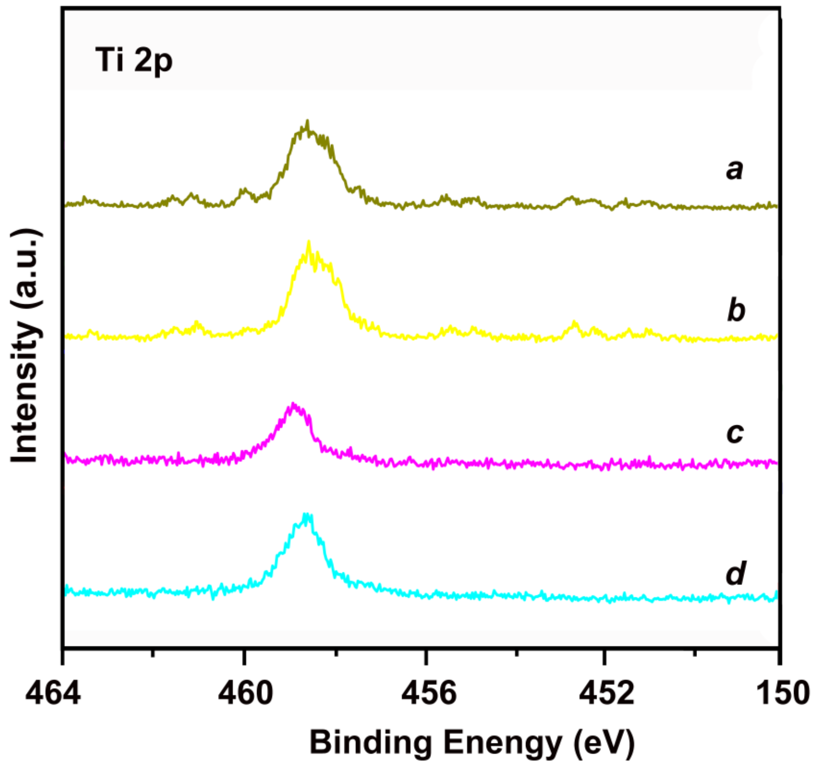

Supplement: Supplementary file 1 [file molecules-29-03462-s001.zip › molecules-3076997-supplementary.pdf]
